# Genetics of Omega-3 Long-Chain Polyunsaturated Fatty Acid Metabolism and Meat Eating Quality in Tattykeel Australian White Lambs

**DOI:** 10.3390/genes11050587

**Published:** 2020-05-25

**Authors:** Shedrach Benjamin Pewan, John Roger Otto, Roger Huerlimann, Alyssa Maree Budd, Felista Waithira Mwangi, Richard Crawford Edmunds, Benjamin William Behrens Holman, Michelle Lauren Elizabeth Henry, Robert Tumwesigye Kinobe, Oyelola Abdulwasiu Adegboye, Aduli Enoch Othniel Malau-Aduli

**Affiliations:** 1 Animal Genetics and Nutrition, Veterinary Sciences Discipline, College of Public Health, Medical and Veterinary Sciences, Division of Tropical Health and Medicine, James Cook University, Townsville, Queensland 4811, Australia; shedrach.pewan@my.jcu.edu.au (S.B.P.); john.otto@jcu.edu.au (J.R.O.); felista.mwangi@my.jcu.edu.au (F.W.M.); richard.c.edmunds@gmail.com (R.C.E.); robert.kinobe@jcu.edu.au (R.T.K.); 2 National Veterinary Research Institute, Private Mail Bag 01, Vom, Plateau State, Nigeria; 3 Centre for Sustainable Tropical Fisheries and Aquaculture and Centre for Tropical Bioinformatics and Molecular Biology, College of Science and Engineering, James Cook University, Townsville, Queensland 4811, Australia; roger.huerlimann@jcu.edu.au (R.H.); alyssa.budd@jcu.edu.au (A.M.B.); 4 Centre for Red Meat and Sheep Development, NSW Department of Primary Industries, Cowra, New South Wales 2794, Australia; benjamin.holman@dpi.nsw.gov.au; 5 Gundagai Meat Processors, 2916 Gocup Road, South Gundagai, New South Wales 2722, Australia; MHenry@gmpgundagai.com.au; 6 Faculty of Veterinary and Agricultural Sciences, University of Melbourne, Melbourne, VIC 3010, Australia; 7 Australian Institute of Tropical Health and Medicine, College of Public Health, Medical and Veterinary Sciences, Division of Tropical Health and Medicine, James Cook University, Townsville, Queensland 4811, Australia; oyelola.adegboye@jcu.edu.au

**Keywords:** Tattykeel Australian White, MARGRA lamb, omega-3 long-chain polyunsaturated fatty acids, fat melting point, intramuscular fat, genetics, meat quality, stearoyl-CoA desaturase, fatty acid binding protein 4, fatty acid synthase, fat metabolism

## Abstract

Meat eating quality with a healthy composition hinges on intramuscular fat (IMF), fat melting point (FMP), tenderness, juiciness, flavour and omega-3 long-chain polyunsaturated fatty acids (n-3 LC-PUFA) content. These health-beneficial n-3 LC-PUFA play significant roles in optimal cardiovascular, retinal, maternal and childhood brain functions, and include alpha linolenic (ALA), eicosapentaenoic (EPA), docosahexaenoic (DHA) and docosapentaenoic (DPA) acids. The primary objective of this review was to access, retrieve, synthesise and critically appraise the published literature on the synthesis, metabolism and genetics of n-3 LC-PUFA and meat eating quality. Studies on IMF content, FMP and fatty acid composition were reviewed to identify knowledge gaps that can inform future research with Tattykeel Australian White (TAW) lambs. The TAW is a new sheep breed exclusive to MARGRA brand of lamb with an outstanding low fat melting point (28–39°C), high n-3 LC-PUFA EPA+DHA content (33–69mg/100g), marbling (3.4–8.2%), tenderness (20.0–38.5N) and overall consumer liking (7.9–8.5). However, correlations between n-3 LC-PUFA profile, stearoyl-CoA desaturase (SCD), fatty acid binding protein 4 (FABP4), fatty acid synthase (FASN), other lipogenic genes and meat quality traits present major knowledge gaps. The review also identified research opportunities in nutrition–genetics interactions aimed at a greater understanding of the genetics of n-3 LC-PUFA, feedlot finishing performance, carcass traits and eating quality in the TAW sheep. It was concluded that studies on IMF, FMP and n-3 LC-PUFA profiles in parental and progeny generations of TAW sheep will be foundational for the genetic selection of healthy lamb eating qualities and provide useful insights into their correlations with SCD, FASN and FABP4 genes.

## 1. Introduction

Sheep production is an important economic activity in many countries because lamb is one of the world’s four major meat classes along with pork, chicken and beef [1]. Sheep are produced mainly for their meat (lamb or mutton) and wool [2]. In 2017–2018, Australia exported 532,000 tonnes carcass weight of lamb representing 61% of total lamb production from 72.1 million head of sheep and was the largest sheep meat exporter in the world worth A$3.28 billion [3]. Apart from sheep meat exports, Australians were also the world’s highest consumers of lamb estimated at 7.3 kg/capita in 2018 [1]. Thus, lamb is a very significant contributor to the Australian economy and a major part of the Australian diet. 

Lamb is a very nutritious, easily digestible and highly valued food with a healthy fatty acid composition [4]. Lamb consumers demand meat that is safe, of consistent eating quality, healthy composition and conveniently easy to prepare [5]. Meat quality is the constitutional standard of lean-to-fat ratio and palatability indices that include visual appearance, aroma, drip loss, colour, texture, pH, intramuscular fat profile, tenderness, flavour and juiciness [6]. The entire processes of feeding culminating in the finishing of animals including their genetic constitution, husbandry practices and handling, all affect the overall quality of meat [7]. There are genuine concerns about high fat consumption, especially fats of animal origin, as their profile has a significant influence on human health because excessive consumption of saturated fatty acids (SFA) is associated with high levels of low density-lipoproteins and cholesterol [8,9]. Both low density-lipoproteins and hypercholesterolemia are predisposing risk factors for cardiovascular disease [10], prostate, mammary and colorectal cancer [11,12], dry eye disease, [13], depression [14], obesity, diabetes [15,16] and neuro-degenerative conditions including Schizophrenia, Alzheimer’s, Parkinson’s disease [17]. In spite of animal lipids being criticised as health-risk factors, it is evident that they actively support many physiological functions and provide health-beneficial omega-3 long-chain polyunsaturated fatty acids (n-3 LC-PUFA) [5]. This is the basis for various animal production strategies aimed at enhancing health-beneficial fatty acids in meat and meat products [18,19]. This is because intramuscular fat, fatty acid content, water holding capacity and consistency largely influence meat organoleptic traits and retail potential namely, juiciness, tenderness, flavour, colour, shelf life and firmness [20,21].

In the quest for a meat sheep breed with good body conformation, superior eating qualities, low fat melting point (FMP), high intramuscular fat (IMF) and healthy n-3 LC-PUFA composition, the Gilmore Family in Black Springs, Oberon, New South Wales, pioneered the development of the Tattykeel Australian White (TAW) breed. This breed was developed over a 15-year period of rigorous breeding, culling and selection of Poll Dorset, Dorper, Texel and Van Rooy rams and ewes with an extensive utilisation of embryo transfer, artificial insemination and natural mating. Although preliminary evidence from our data in Table 1 suggests that the TAW sheep breed exclusive to the MARGRA brand of lamb has an outstanding low FMP, high n-3 LC-PUFA content and IMF, comprehensive peer-reviewed publications on its eating quality attributes and n-3 LC-PUFA profile are currently lacking. This necessitates further research into genetic factors that may determine IMF, FMP, n-3 LC-PUFA in this breed. Many genes and enzymes are responsible for fatty acid metabolism and their correlations with meat quality traits. However, the roles of stearoyl-CoA desaturase (SCD), fatty acid binding protein 4 (FABP4), and fatty acid synthase (FASN) are the most critical [22,23] and need further elucidation herein. Therefore, the primary objective of this review was to critically appraise the published literature regarding fatty acid synthesis and metabolism, IMF, FMP, and carcass quality to identify knowledge gaps and highlight research opportunities associated with nutrition–genetics interactions influencing n-3 LC-PUFA that can inform future meat-eating quality investigations in TAW lambs.

## 2. Fatty Acids, Classifications and Functions

Lipids are preferentially utilised as the major energy source in enteral diets owing to their high caloric value [24]. Fats are triglycerides comprising glycerol and fatty acids. Apart from their main biological function of energy storage, lipids are essential components of cellular membranes and signalling molecules [25]. Thus, Patterson et al. [26] stated that fatty acids as the “building units” of lipids, are hydrocarbon chains having a carboxyl (-COOH) group at one end and a methyl (-CH3) group at the other. When three fatty acids are attached to a glycerol molecule, energy-storing triacylglycerols are formed [27]. The amphiphilic structure of fatty acids arising from their hydrophilic carboxyl group attachment to a hydrophobic hydrocarbon chain or tail provides the ideal energy storage powerhouse that is characteristic of triacylglycerols [21]. The bonds between the carbon atoms in a hydrocarbon chain differentiate between saturated (SFA) and unsaturated (UFA) fatty acids. However, SFA consist of less reactive single bonds only, while UFA have one (monounsaturated, MUFA) or at least two (polyunsaturated, PUFA) reactive double bonds. Fats containing significant levels of MUFA like oleic acid (C18:1), contribute to high quality meat due to low melting point which leads to favourable meat flavour, tenderness and juiciness [28]. C18:1 as the most abundant MUFA in the adipose and muscle tissues of ruminants, and it is not easily susceptible to oxidation [29]. PUFA are further divided into four families: omega-3 (n-3), omega-6 (n-6), omega-7 (n-7) and omega-9 (n-9), based on the position of the initial double bond on the methyl terminal [30] or the location of the last double bond relative to the terminal methyl end of the molecule [31]. 

Fatty acids can also be subdivided into essential and non-essential fatty acids. The latter can be synthesised de novo (mainly in the liver), without the need for dietary supplementation [32] while the former on the other hand, cannot be synthesised by mammals and need to be included in the diet [21]. Essential fatty acids play significant roles in enzymatic regulation, eicosanoid synthesis, cell signalling, control of neuronal migration, neuromodulatory and neurotransmitter activities [33,34]. Some deficiency symptoms of essential fatty acids have been identified in a number of nutrition-related complications in the liver and kidneys, especially in children, to include dry and flaky skin, diarrhoea, anaemia, stunted growth and poor wound healing as well as compromised immunity leading to secondary infections [35]. Therefore, it is important to supply this group of fatty acids in correct proportions right from conception, throughout pregnancy and infancy.

### 2.1. Omega-3 Long-Chain Polyunsaturated Fatty Acids

The word “omega” (ω) in relation to fatty acids denotes the terminal carbon atom furthest from the functional carboxylic acid group (-COOH). These structural differences confer unique individual functions. Omega-3 (n-3) long-chain polyunsaturated fatty acids are a family of PUFA made up of α-linolenic acid (ALA, C18:3n-3), a precursor for the more functionally potent longer chain Eicosapentaenioc acid, 20:5n-3 (EPA) and Docosahexaenoic acid, 22:6n-3 (DHA) members of the family [36,37,38]. Omega-3 PUFA increase the stability of cell membranes, regulate immune function, block excessive inflammatory reaction [39], reduce systemic inflammatory response syndrome, various organ dysfunction syndromes, infectious complications and depress tumour growth [28,40]. The most important functional n-3 LC-PUFA related to human well-being are EPA and DHA [41]. Furthermore, the hitherto neglected roles of docosapentaenoic acid (DPA; 22:5n-3) are currently evolving [42,43]. A number of research findings have established that n-3 LC-PUFA are potent therapeutic agents for the suppression of inflammation, thus playing critical roles in a number of inflammatory conditions including diabetes, artherosclerosis, asthma and arthritis [34,44].

Cardiovascular ailments and cancer are the main causes of human death globally [45,46,47]. Thus, consumption of n-3 LC-PUFA decreases the danger of cardiovascular diseases by depressing systolic resting heart rate, diastolic blood pressure [48], blood viscosity [49], plasma fibrinogen [50] and platelet aggregation [51]. They also improve blood vessel function [52]. In adults, increased intake of n-3 LC-PUFA has remarkable brain health benefits, reduced risk of dementia and late cognitive malfunction [53], overall health at pregnancy [54], insulin resistance [16], depression and retarding the progression of certain cancers [55,56]. Gould et al. [57] reported that n-3 LC-PUFA play significant roles in neural development in embryos and at infancy. High consumption of EPA and DHA has also proved useful in improving foetal brain, retinal development, and reducing the risks associated with cardiovascular and Alzheimer’s diseases [53]. Welch et al. [57] proposed DHA, EPA, n-3, ALA and LA dietary intakes of 0.16, 0.11, 1.50, 1.23 and 12.35g/d for men and 0.13, 0.09, 1.22, 0.99 and 9.42 for women, respectively. It has been recommended that patients susceptible to coronary heart disease should consume at least 1g of DPA and DHA daily; and good sources of these nutrients include seafood, particularly fatty fish (for example, mackerel, herring, sardines, salmon, trout, kippers, pilchards, eels and tuna), whales, seals and oil supplements from fish, cod liver, krill and algae [58,59]. However, the use of marine fish oil has some drawbacks including typical fishy smell, unpleasant taste, expensive cleansing procedure and adulteration by environmental contaminants including radioisotopes, dioxins and heavy metals [60,61]. Western diets contain 1.5–10.0 g of n-6 fatty acids which are derived from plant oils rich in linoleic acid. ALA is also found in canola (rapeseed) oil, flaxseed (linseed) oil, rapeseed oil, soybean oil, pumpkin seeds and walnut oil [62,63,64]. However, humans lack the enzymes required to transform n-3 from n-6 fatty acids, they also have a limited capacity to elongate and change ALA to EPA and DHA [63].

**Figure 1:**
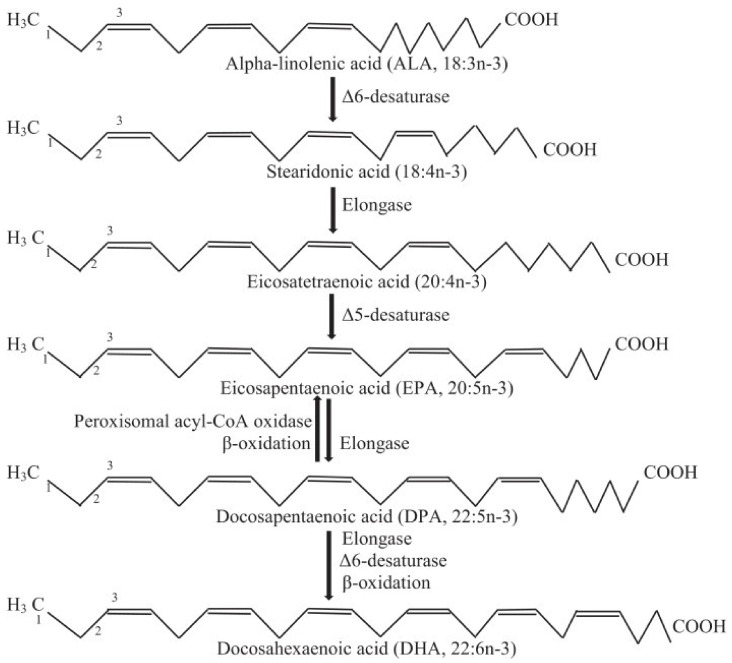
Pathway for the biosynthesis of omega-3 long-chain polyunsaturated fatty acids (n-3 LC-PUFA) from α-linolenic acid (ALA) [59].

Figure 1 depicts the pathway where EPA is produced from simpler, plant-sourced n-3 fatty acids like ALA (18:3n-3) [59]. The enzymes involved in n-3 fatty acid interconversion are identical with the analogous n-6 fatty acid pathway for the transformation of linoleic acid (18:2n-6) to arachidonic acid (20:4n-6). Majority of these processes involve the addition of a double bond between two carbon atoms (desaturation) and addition of two carbon atoms (elongation reactions) [59,64]. While the enzymes involved in elongation and desaturation pathways are fairly understood in monogastrics, their roles in the interconversion of n-6 to n-3 fatty acids in ruminants is less understood, especially in Tattykeel Australian White lambs, due to biohydrogenation. This represents a major knowledge gap.

Kris-Etherton et al. [65] reported that even though fish oil remains an excellent source of EPA and DHA, Lum et al. [66] recommended that attention is increasingly shifting towards cheaper but equally good substitute sources of n-3 fatty acids, including microalgae known to have high elongase and desaturase enzyme activities necessary for the biosynthesis of EPA and DHA [67]. DPA (C22:5) is similar to EPA with the same number of double bonds but has two more carbon chain units [68]. Its functions were in the past, poorly understood, but currently unravelled [43]. Epidemiological trials in humans have demonstrated high levels of DPA to be favourably correlated with lesser blood triglycerides, cholesterol, inflammation and a reduced total risk of cardiovascular diseases and acute myocardial infarction [46,69,70,71]. DPA is an active and potent stimulator of endothelial cell migration, an important part of the embryonic vascular system [72]. It also acts as a precursor for the synthesis of resolvins which are neuroprotective in function [43]. In other studies, Phang et al. [73] demonstrated that when applied to platelets or PC-21 human epithelial cell lines, purified DPA reduces platelet accumulation and aggregation more efficiently than EPA and DHA [74] and leads to endothelial cell migration [75] and inhibition of chronic inflammation [76].

#### Fatty Acid Profile and Nutritional Value 

The fatty acid profile of meat is related to meat quality sensory attributes, nutritional value and health benefits [18,77]. For instance, a direct relationship between the content of stearic acid in the fat and fat hardness exists, because as the content of stearic acid increases, so does the fat hardness. This in turn, influences marbling fat melting point and meat juiciness. The quantity and type of intramuscular fat and fatty acids in both muscle and adipose tissues influence eating quality, juiciness, tenderness, flavour, colour, shelf life and firmness of meat [20,21,78]. Fat content and amount of fatty acids are quantified in mg/100g of meat [79], whereas human nutritionists assess nutrient value of food per 100 g of serve. For food to be categorised or claimed as a source of n-3 LC-PUFA in Australia and New Zealand, its EPA and DHA contents should be greater than 30mg per serve, and declared a good source if it has at least 60mg of EPA and DHA for each standard serve [79,80,81]. In Europe, it is 40mg per 100g [82]. The World Health Organization [83] recommended that daily fat intake should be 30% of total energy, and of this, SFA should be reduced to 300mg per day. They also advocated that a reasonable balance of fatty acids in food should be established where intakes of cholesterol and SFA are decreased. The ratios between SFA and PUFA and n-6 and n-3 fatty acids determine the nutritional value of meat [78]. However, Simopoulos et al. [84] documented that in developed and industrialised countries, there is growth in the consumption of SFA, n-6 PUFA and trans fatty acids and a marked reduction in n-3 PUFA intake. The diets in these parts of the world have an n-6:n-3 PUFA ratio of about 15:1, compared to an ideal recommended ratio of 4:1 [51,85]. This unbalanced consumption leads to low tissue levels of DHA and EPA [86], resulting in higher incidences of inflammatory processes, cardiovascular diseases, obesity, inflammatory bowel disease, rheumatoid arthritis and cancer [87].

### 2.2. Factors Affecting Fat Profile in Ruminant Muscle and Adipose Tissues

Fatty acid profile is influenced by biohydrogenation in the rumen, dietary concentrate supplementation versus pasture finishing and genetics [17,88].

#### 2.2.1. Biohydrogenation 

Sheep, like all other ruminants, harbour a diverse microbial population in their rumen that enables the digestion of complex plant materials into more absorbable nutrients [89]. The rumen ecosystem is composed of anaerobic bacteria, protozoa, fungi, methanogenic archaea and phages [90]. Microbes play different, yet complimentary, roles in the rumen. Bacteria enzymatically convert sugars to volatile fatty acids (acetic 60–70%, propionic 15–20% and butyric acids 10–15%), which are the main energy substrates for ruminants [91,92]. Protozoa on the other hand, degrade complex carbohydrates and nitrogen into nutrients that are made available to the host, while anaerobic fungi engage in cellulolytic degradation activities [93]. The type and amount of fat delivered to the rumen [94], temperature of 38–39 °C [95] and pH range between 6.0 and 6.7 [96] dictate optimal rumen microbial function. 

Ruminant diets are commonly made up of forages and concentrates with fats sometimes included, to raise the energy level in rations for lactating females or to enlarge the amount of human-health beneficial n-3 LC-PUFA, and bioactive conjugated linoleic acid in meat and milk [97]. Upon entry into the rumen, ingested lipids are degraded by microbial lipases via lipolysis [93,95,98]. Lipolysis breaks down lipids and releases free fatty acids from esters, thus facilitating biohydrogenation where the number of double bonds is reduced on the carbon chain [95], or under ideal conditions, 85% of esterified dietary lipids in the form of galactolipids, phospholipids and triacylglycerols are hydrolysed [95,99]. UFA get converted into SFA in the rumen due to microbial biohydrogenation activities involving series of consecutive conversion pathways leading to an abundance of fatty acid isomers [97], and remain a major human public health issue [100]. The bulk of the dietary fatty acids are 18-carbon UFA (linolenic acid, 18:3n-3; linoleic acid, 18:2n-6 and oleic acid, cis-9 18:1) [101]. However, the major biohydrogenation intermediate product in a ruminant fed forage diet is trans-vaccenic acid (trans-11 C18:1, t-VA) [102]. t-VA acts as a precursor required for the production of SFA in the rumen to yield stearic acid (C18:0). Conversely, it is desaturated by Δ9-desaturase enzyme in the mammary gland to yield cis-9, trans-11 C18:2 and its CLA isomer that can be easily detected in milk and meat [103]. Other end products of rumen metabolism are carbon dioxide, methane and traces of hydrogen [104] used as energy sources for the reduction of carbon dioxide to methane [105]. Short chain fatty acids (acetic acid (C2), propionic acid (C3), and butyric acid (C4)) produced are absorbed, transported and metabolised by different organs in the body of the host animal while carbon dioxide and methane are expelled from the body through different cycles of eructation or belching [106].

#### 2.2.2. Influence of Concentrate or Forage Finishing on Lamb Performance and Meat Quality

Lamb finishing on pasture is cheaper than grain feeding [107], but the viability of pasture-finishing depends on a consistent supply of good quality forage [108]. This is achieved by growing a mixture of grasses and legumes. Legumes increase the nutritional quality through higher digestibility and protein content [109,110], healthier fatty acid composition and increased oxidative stability [111]. Meat derived from pasture-finished animals has higher CLA and PUFA content especially of the n-3 series in the *longissimus thoracis et lumborum* muscle than meat from feedlot or grain-fed ruminants and at the same time, the proportion of fat and cholesterol in meat from grass-fed ruminants is lower [19,112,113]. Mixed pasture finishing improves growth performance and carcass traits of grazing ruminants [114]. Apart from contributing to landscape maintenance, nature preservation, pasture feeding system is generally desired by health-conscious organic meat consumers [5]. However, pasture-finished meat has some limitations that include lower carcass weight [115] and extended periods of feeding to attain market weight specifications compared to their contemporaries finished on grains [114,116,117]. Furthermore, grain finishing gives higher attainment of desired weights, better meat quality with regard to tenderness, marbling, ribeye area (REA) and backfat thickness (BFT), higher stocking rate per land unit than their counterparts finished on pasture [116,118]. In a grain finishing system, the net energy and glucose available for fat synthesis as muscles grow, reduce in older animals, and this leads to a higher fat content than obtained in a grass finishing system.

The biochemical processes outlined above are influenced by genetic differences and particularly enzymes and genes involved in fat metabolism. There are no reference materials in peer-reviewed sources on how all these biochemical processes relate to the TAW.

## 3. Lipogenic Genes and Associations with Genetic Selection for Meat Quality 

Routine phenotypic data collection may be an arduous task given that live-animal proxies hardly exist for meat quality traits and the related costs of such data collection are high [119], ranging from Au$50 to 100 per animal. Genomic data therefore is significant in the design and implementation of animal breeding and improvement programmes to rapidly increase the frequency and potency of desirable genes in the population [120,121]. The utilisation of genomic data can raise the accuracy level of estimated breeding values (EBV), thus increasing the rate of genetic progress [122,123,124]. Progressive advancements in molecular genetics have resulted in an increased identification and documentation of genes or markers influencing meat quality traits [125]. Casas et al. [125] reported that DNA polymorphisms in some identified candidate genes were associated with meat tenderness. Genomic selection involves decisions that are focused on breeding values utilising genome wide markers such as SNP [124]. In sheep production, genomic prediction offers reliable alternatives because many traits influence fatty acid inheritance. Accurate genomic estimated breeding values (GEBV) for these traits would lead to greater genetic gains [122]. GEBV calculation is dependent upon the reference population that has been determined for the trait and genotyped for the markers [122]. Hayes et al. [126] established the fact that the degree of accuracy of GEBV on selection candidates rests on the proportion of this reference population and the level of the linkage disequilibrium between SNPs and quantitative trait loci (QTL). Traits that are difficult or expensive to measure are quite challenging to get large reference populations for accurate GEBV prediction [122].

SNPs in a number of genes can influence the fatty acid profile of ruminants [127], however, SNPs in FASN, SCD and FABP4 would be considered in this review because of their critical roles in fatty acid metabolism. Furthermore, Bhuiyan et al. [128] reported five SNPs in the FASN gene in cattle, and one of the SNPs was correlated with the composition of lipids, and may be utilised as a marker in breeding programs. However, in sheep, there is paucity of information on these genes. From the literature, there are research attempts aimed at linking the lipid profile of lambs with SNP [129], but to our current knowledge, there is no published information on any identified SNP in TAW sheep. This represents a major knowledge gap.

### 3.1. Stearoyl-CoA Desaturase (SCD) 

The SCD gene encodes for delta-9 desaturase enzyme. It is an iron-containing endoplasmic reticulum enzyme [130,131], that catalyses a rate-limiting step in the conversion of SFA into MUFA in mammalian adipose cells [131,132]. The principal product of the desaturase enzyme is oleic acid, which is formed by the desaturation of stearic acid [133]. In cattle, the SCD gene comprises two isoforms; SCD1 and SCD5 [134]. The SCD1 gene, mapped on bovine chromosome 26, codes for stearoyl-CoA desaturase [130]. The fatty acid profile of stored fat reflects the earlier action of SCD on substrates such as palmitic or stearic acids [135]. Similarly, Smith et al. [136] reported that there are three fatty acid desaturases in animal tissues: Δ5, Δ6, and Δ9 desaturases, and that of these, only Δ9 desaturase acts upon SFA to convert them to their respective MUFA. It serves as a catalyst in the synthesis of UFA by incorporating a *cis*-bond between the 9th and 10th carbon atoms of FA with chain lengths of 10-18 carbons in adipose tissues and mammary glands [137]. Other researchers agree that the SCD enzyme is essential in the biosynthesis of MUFA such as oleic (C18: 1n-9) and palmitoleic (C16: 1n-9) acids, formed after the addition of a double bond in the Δ9 position of their precursors, C18: 0 and C16: 0 fatty acids [138]. It has been documented that SCD converts C18:1 trans-11 to C18:2 cis-9, trans-11, said to be correlated with anticarcinogenic and antiatherogenic effects [139,140]. It also increases the ratio of MUFA to SFA [141]. In sheep milk, this gene encodes the SCD enzyme found in a locus where a positional QTL has been identified for the CLA:VA ratio [142]. 

In sheep, an SCD SNP (SCD5, rs423661926) was found to be significantly associated with rib eye area and genotypic effects ranged from 0.035 to 0.923 [143]. The SCD gene has also been reported to harbour polymorphisms that affect milk fat content, specifically, palmitoleic acid, LA, VA, SFA and MUFA and ratios of n-6:n-3 and palmitoleic acid:palmitic acid [144]. The expression of this gene is controlled by the diet (particularly its content of n-6 and n-3 PUFA), environmental and hormonal factors [145]. In sheep, as reported by Dervishi et al. [146], grazing raises the quantities of CLA, total PUFA and n-3 PUFA in lamb, which is a favourable and desirable option in line with health-beneficial human dietary guidelines [141].

### 3.2. Fatty Acid Synthase (FASN) 

FASN is the gene encoding for fatty acid synthase enzyme and is a versatile and valuable protein complex that controls the de novo biosynthesis of long chain fatty acids [147]. According to Chirala et al. [148], this gene plays essential roles during embryogenesis and adulthood fatty acid synthesis. Zhang et al. [149] demonstrated associations arising from meat fatty acid profile and FASN candidate gene polymorphisms. In bovine species, the FASN gene has been mapped on BTA19 where many QTL influencing beef fatty acid profile, adipose and milk fat contents were found [150,151]. The four exons (39–42) in the FASN complex which encode for the thioesterase (TE) domain are accountable for the synthesis of fatty acids, especially C16:0, by hydrolysing the acyl-S-phosphopantetheine thioester. Consequently, Zhang et al. [149] observed that the TE domain dictates the product chain length of FASN and variability in the TE domain amongst individuals is said to be a heritability candidate for variability in fatty acid profiles. Roy et al. [151] found higher bovine FASN expressed in a number of tissues and organs especially the brain, testis and adipose tissue and less in liver and heart and FASN assists in catalysing the reaction steps involved in the transformation of acetyl-CoA and malonyl-CoA to palmitic acid. Similarly, FASN gene uses Malonyl CoA and Acetyl CoA as substrates, while NADPH acts as a co-factor [152]. The FASN action in mammals largely yields C16:0 with negligibly minute levels of C14:0. 

In humans, Chakravarty et al. [153] reported that the TE domain possesses a hydrophobic groove which contributes the fatty acyl substrate binding site with high specificity regarding C16-acyl ACP, but not C14-acyl ACP. Oh et al. [154] demonstrated a favourable impact of FASN gene on fatty acid profile. FASN is a versatile and important protein complex which catalyses the synthesis of long-chain SFA. However, the differences in TE domain (that is exons 39–42, that account for fatty acid synthesis termination of the FASN gene), would be a candidate for heritable differences in fatty acid profile [154]. To our current knowledge, apart from the short communication of Sanz et al. [155] that identified novel polymorphisms in the 5′UTR of FASN, PROP1, GPAM, MC4R, FADS and PLIN1 ovine candidate genes and their relationships with gene expression and diet in a study with Spanish sheep (Rasa Aragonesa, Roja Mallorquina and Assaf), Chinese Sunit sheep [156], and New Zealand sheep [157], there is no literature published on FASN-FADS-PROP1 genes and their correlations with growth and meat quality traits in TAW or any other sheep breed, thus presenting a significant knowledge gap.

### 3.3. Fatty Acid Binding Protein4 (FABP4) 

To date, nine sub-types of fatty acid binding proteins (FABP) can be identified *(FABP1–FABP9)* and are named based on the tissues they are found in highest concentration [158]. FABP4 is also known as adipocyte fatty acid binding proteins (A-FABP or aP2). The location of FABP4 gene varies with livestock species; for instance, in sheep and cattle, it is located on chromosomes 9 and 14, respectively [158]. FABP4 encodes for a group of fatty acid binding proteins and is abundantly expressed in the adipose tissue where these binding proteins are important in glucose homeostasis, FA metabolism, transport and absorption, by their association with peroxisome proliferator-activated receptors (PPAR) [159,160]. Apart from differences in two regions of ovine FABP4 in lean and fat selection lines of Coopworth [161], Romney [162], and Rasa Aragonesa [163], breeds, published reports on the FABP4 gene in sheep are few and scanty. 

Most of the reported studies on FABP4 gene have been in beef cattle. Barendse et al. [164] reported that a splice site SNP of the FAPB4 gene appeared to be connected with the deposition of IMF in the *Longissimus thoracis et lumborum* muscle. In terms of variation in the FABP4 gene, Yan et al. [162] found that it is linked with growth, deposition of fat and carcass traits. In Japanese black cattle, Hoashi et al. [165] documented a relationship existing between FABP4 and fatty acid profile, while Ardicli et al. [166] associated SNPs in bovine FABP4 with escalation in live weight, chilled carcass weight, marbling score and back-fat thickness, but without any colour differences or carcass dimension measurements. In Aberdeen Angus and Blonde d’Aquitaine cattle, the FABP4 SNP 7516G>C was analysed for association with IMF composition of the *Longissimus thoracis et lumborum* muscle between the 12th and 13th ribs. In Angus cattle, the CC genotype was reported to be 52% and 64% lower in myristoleic acid, and 33% and 35% lower in LA than CG and GG genotypes, respectively. On the other hand, in Blonde d’Aquitaine cattle, the CC genotype had elevated levels of arachidonic acid and EPA, and comparatively less oleic acid and total SFA than CG genotype. The GG genotype was only detected in one cow [167]. In Wagyu × Limousin crosses, the g.7516G>C SNPs were investigated for any existing relationship between marbling score and depth of subcutaneous fat. An association was established between CC genotype with lower marbling and fat depth. While GC genotype recorded the highest scores, GG genotype was intermediate [160]. Furthermore, in Korean Native cattle, FABP4 SNPs had a correlation with backfat thickness [168].

In sheep, FABP4 plays an important part in glucose and lipid metabolism in adipocytes [169,170]. Therefore, FABP4 polymorphisms are believed to have a significant influence on live performance and carcass characteristics [162,171], meat tenderness, marbling score and IMF content in sheep. For instance, in Romney sheep, [156,157] reported five variants (A1 − E1) in region-1 (exon 2 − intron 2) and three variants (A2 − C2) in region-2 (exon 3 − intron 3) wherein A1 was associated with a decrease in leg, loin and total meat yield, while A2 was associated with a decrease in weaning weight and pre-weaning growth rate. Haplotype A1-A2 was found to be associated with a decrease in birth weight, pre-weaning growth-rate, hot carcass weight, loin meat yield, shoulder meat yield and total meat yield, while haplotype A1-B2 was associated with increased fat depth at the 12th rib (V-GR). Taken together, their finding supports the contention that variation in FABP4 affects growth and meat production. To our current knowledge, nothing is known about the FABP4 gene in TAW breed and this major knowledge gap needs to be filled by researchers. 

### 3.4 Other Fat Related Genes

Several other genes reported to be associated with fat are cocaine- and amphetamine-regulated transcript (CART) with *Longissimus thoracis et lumborum* muscle IMF content [172]. The genes encoding leptin are associated with backfat thickness and marbling score [173], while the gene encoding diacylglycerol O-acyltransferase (DGAT1) is associated with liveweight, fat thickness, rib-eye area and shoulder weight in Texel lambs [143] and IMF [174]. The growth hormone 1 (GH1) gene is weakly correlated with rump fat [175] and sterol regulatory element-binding protein 1 (SREBP1) has been reported to be correlated with FA profile [128]. However, all these studies were in cattle. Similar investigations in TAW have not been published and represent major research knowledge gaps. An updated summary of candidate genes associated with meat quality in livestock [176] is shown in Table 2:

## 4. Meat Eating Quality

Meat eating quality is influenced mainly by marbling, juiciness, tenderness and flavour [197]. Studies with lamb have shown that carcass intramuscular fat deposition and FA composition account for eating quality variation [198,199]. Consumption of lamb IMF is important to humans since it helps with the delivery and absorption of fat-soluble vitamins, and exerts positive effects on immune response [200] as exemplified by Calder’s work [58] demonstrating the relationship between fatty acid composition of immune cells and their function. Marbling score to date remains one of the most important traits and reason why carcass evaluation is carried out in the abattoir [201]. In the United States of America for instance, it is the major index considered in assigning beef quality grades [202] because the quantity and distribution of IMF in the longissimus muscle area have marked effects on tenderness, flavour, juiciness and colour [203]. The amount of IMF is greatly influenced by a number of factors. These include animal age and breed, weight at slaughter [204], diet [205] and growth rate [206]. Adipogenesis in the animal’s life commences with deposition of visceral fat, subcutaneous, intermuscular and intramuscular fat occurs last [207]. 

Deposition of IMF is a highly heritable trait and is positively correlated with overall body fatness [203]. Nutritional value is an essential determinant of meat quality. Hocquette et al. [208] reported that awareness amongst consumers has greatly increased over the years regarding the relationships that exists between diet, health and well-being which has resulted in selection of foods which are healthier and nourishing. Level of marbling, fatty acid composition, biological value of protein, minerals and vitamins are essential elements of nutritive value of any food [209]. 

### 4.1. Influence of IMF on Lamb Eating Quality

#### 4.1.1. Tenderness

Meat tenderness has been identified as the most important sensory trait consumers consider when making decisions to purchase meat [210] as it probably affects consumers’ understanding of acceptability. They are prepared to pay a premium for consistently tender meat and other traits they value [211]. Meat tenderness affects the profitability of the lamb meat industry. It depends on a number of factors including muscle sarcomere length, integrity, connective tissue content and composition [212]. Meat tenderness differs within and between animals and the different muscles [213] and is influenced by age of the animal, its sex, breed, genotype, nutrition, ante-mortem stress and post-mortem handling [214]. Refrigerating carcasses just after slaughter leads to intense contraction of the fibre muscles known as “cold shortening” which is an undesirable trait [215]. Cold shortening is the result of the rapid chilling of carcasses immediately after slaughter, before the glycogen in the muscle is converted to lactic acid. With glycogen still present as an energy source, the cold temperature induces an irreversible contraction of the muscle, thus impacting negatively on tenderness. Perlo et al. [216] reported that meat from lambs finished on forage-based diets was less tender than meat from their counterparts fed concentrates. In contrast, Sanudo et al. [217] reported that meat from grazing animals was more tender than from concentrate-fed lambs. This difference could be due to variation in carcass fatness resulting in differential cooling rates during rigor development. Furthermore, the use of fatness measures as covariates during statistical analysis can provide an unbiased basis for treatment comparison to judge if the observed differences are solely due to intrinsic dietary influences [218]. Meat from young lambs is more tender, has lower fat content and preferred by most consumers compared to mutton from older sheep [219]. The metabolic processes of lipogenesis, lipolysis and fatty acid transport culminate in IMF deposition [220]. Therefore, a diet with high-energy content leads to more lipogenesis [221]. Furthermore, the level of intramuscular fatty acids is mainly regulated by either inducing or inhibiting genes encoding for specific metabolic enzymes normally linked with lipid metabolism or transcription factors [222].

Tenderness is a proclamation of meat texture and is regarded as a major sensory quality attribute that is related with consumer satisfaction and positively correlated with juiciness and flavour, with consumers willing to pay more for tender meat [223]. It is closely related to meat structure, biochemical activity as well as time that elapses between slaughter and consumption [224]. Ali et al. [225] reported that meat tenderness is influenced by the rate and level of glycolysis and the onset of rigor post-slaughter. According to Starkey et al. [226], meat tenderness is dependent on intrinsic physiological traits of the live muscle and processing elements developed after rigor, while Rhee et al. [227] attributed it to sarcomere length. Sarcomere length governs the overall length of muscle fibres and plays a significant role in the mechanical structure of muscles [228]. 

#### 4.1.2. Flavour

Flavour is mainly as a result of volatile substances that impact strongly on the sensory characteristics of red meat [229]. Meat flavour is affected by animal breed, nutrition, genotype, temperament, aging after slaughter, cooking method and their interactions [229,230]. Meat flavour is derived through cooking, as raw meat possesses slight or no aroma. During cooking, a number of complex reactions are observed between a number of non-volatile compounds of lean and fatty tissues making the meat flavoursome [231,232]. The major reactions seen in aromatic volatile production are the Maillard reactions between amino acids and carbohydrates and heat degradation of fats [233]. Gylcosylamine which is a product of condensation of amino compounds with carbonyl group of reducing sugar precipitated by heat, becomes dehydrated to yield furfural, furanone, hydroxyketones and dicarbonyl compounds [231]. However, these results of Maillard reactions arising from interaction linking carbohydrates and proteins contribute significantly to meat flavour [234]. In sheep, lamb and mutton have a distinct strong species-related flavour that is influenced by various antemortem and postmortem factors such as pH, age, sex, diet, type of cooking, and curing. Post-cooking storage and modulation of lipid oxidation in mutton also has effects on flavour characteristics and various chemical compounds have been implicated as responsible for or contributing to ovine flavour [232,234]. Of those compounds, medium-length branched-chain fatty acids are the most important. Although processing methods that reduce or modify species flavour such as washing and extrusion with non-meat ingredients have been evaluated, definitive generalisations regarding sheep production management practices yielding meat with the most desirable flavour attributes have not yet been made [232]. 

#### 4.1.3. Juiciness 

Juiciness is an organoleptic index of the quantity of moisture released from meat and the degree of salivation during the process of mastication [235]. Meat juiciness is dependent on water and fat contents [236]. de Lima et al. [237] reviewed the intrinsic factors affecting sheep meat quality and reported that the level of marbling affects different sensory attributes, especially juiciness. Cloete et al. [238] evaluated sheep breeds and reported that the lower proportion of IMF in meat from Merino breed was responsible for lower sensory score for initial juiciness and lasting succulence compared to other sheep breeds. This therefore explains why juiciness has a positive correlation with water holding capacity as well as level of intramuscular fat in meat as demonstrated by the work of Hocquette et al. [239] showing that IMF has a profound effect on juiciness and flavour. Human perception of juiciness is elevated as the IMF level in meat increases [240]. In general terms, juiciness is more of a sensory trait for pork than flavour and tenderness [241] while beef consumers rate tenderness higher [242].

### 4.2. Fat Melting Point 

The hardness or softness of fat is determined by its melting point. Flakemore et al. [199] stated that soft fat has a comparatively lower melting point than hard fat and this has implications for meat processors in abattoirs. MUFA are characterised by lower melting points than SFA, an attribute that favours meat flavour, tenderness and juiciness [28]. Fat melting point is affected by the physical and chemical structures of fatty acids, which in turn, drive carcass evaluation, classification and sensory characteristics of meat [243]. Furthermore, fat melting point is influenced by the molecular weight, the number and configuration of double or triple bonds in the fatty acid structure [244]. Red meat consumers prefer fats with low melting point [199,245] because of their association with reduced risks of cardiovascular diseases [246]. Hard fats pose meat processing and safety challenges in the boning room [247]. In sheep, Holman et al. [205] reported FMP in Merino, Dorset, Black and White Suffolk breeds ranging from 41.5 to 44.8 °C. FMP ranging from 40.6 to 48.0 °C have also been reported in Dorset, White Suffolk and Merino breeds [199]. However, published data on FMP in the TAW sheep breed are currently not available. 

## 5. Conclusions and Future Research

The TAW is a new breed. Mechanisms explaining the impacts and expression patterns of genes associated with intramuscular fat, fat melting points and n-3 LC PUFA on meat sensory attributes are neither currently published nor fully understood. Future work should attempt to unravel single nucleotide polymorphisms, expression patterns and molecular mechanisms of various fat related genes and growth responses of TAW lambs to diverse feedlot finishing diets with and without omega-3 oil inclusion.

Specific knowledge gaps include:

- Early selection decision tools for meat quality traits in TAW lambs are currently non-existent. Most reported selection programmes on fatty acid profile and meat quality traits in other sheep breeds are based on carcass data after the animals have been slaughtered and long gone. Pioneering studies using biopsy sampling of the *Longissimus* muscle in rams, ewes and lambs to directly determine n-3 LC-PUFA, IMF and FMP contents while the animals are young and alive for early selection and breeding purposes are needed.
- Published data on how parents selected for their high n-3 LC-PUFA, IMF and low FMP pass these genes to their offspring are currently non-existent in the TAW breed. Pioneer studies to estimate heritability values based on actual performance data and not estimated breeding values are recommended.
- SCD, FASN and FABP4 genes have been documented to exert some influence on carcass fat traits in other bovine and ovine breeds. No such data exist for the TAW breed. There is the need to sequence the FABP4, FASN and SCD genes to provide foundational data underpinning their roles in fatty acid metabolism unique to the TAW breed.
- In-depth feedlot growth studies are required for better understanding of the interactions between n-3 LC-PUFA oil diets, finishing performance, and carcass traits of TAW lambs to afford industry players the opportunity to utilise them for greater economic gains.
- A cost–benefit analysis of the implication of including n-3 LC-PUFA rich oil in feedlot finishing diets will be of immense industry significance to lamb producers, feed millers and meat processors.

## Figures and Tables

**Table 1:** Tattykeel Australian White carcass and meat quality characteristics (*n* = 217).

Trait	Mean ± SE	Range
Fat melting point (°C)	34.08 ± 1.4	28.0–39.0
Intramuscular fat (%)	4.4 ± 0.2	3.4–8.2
Hot standard carcass weight (kg)	24.6 ± 2.7	19.5–30.7
Dressing percentage	50.2 ± 2.2	47.0–54.4
Fat score	4.7 ± 0.6	4–5
GR fat depth (mm)	16.4 ± 3.5	10–24
Tenderness (N)	32.3 ± 5.1	20.0–38.5
pH	5.63 ± 0.11	5.53–6.83
Overall consumer liking (9-point scale)	8.2 ± 0.9	7.9–8.5
Omega-3 long chain PUFA (mg/100g)		
EPA (20:5n-3)	24.3 ± 5.2	17.8–44.8
DHA (22:6n-3)	8.3 ± 2.7	3.4–12.1
DPA (22:5n-3)	25.2 ± 8.0	14.0–80.3
EPA + DHA	32.6 ± 7.0	33.6–69.9
EPA + DHA + DPA	57.9 ± 13.6	49.1–132.5

EPA, eicosapentaenoic acid; DHA, docosahexaenoic acid; DPA, docosapentaenoic acid.

**Table 2:** Candidate genes associated with meat quality traits in livestock.

Animal	Candidate Genes	Traits	References
Sheep	CAST MSTN FADS2, ELOVL2, SCD, CPT1α, SREBF-1 FABP4 MYF5 Callipyge	Carcass Carcass, meat quality Fatty acids Carcass yield Leg and loin yield Muscular hypertrophy	[177] [178] [179] [161,162] [180] [181]
	GDF8 FAD CAST FASN, FABP4, DGAT1, SCD	Muscular hypertrophy Omega-3 long-chain PUFA Tenderness Fat metabolism	[182] [183] [184] [146,163]
Cattle Pig	FABP4, SCD, PPAG, ACACA, LPL CAST Leptin/Thyroglobulin Myostatin DGAT_1_ HAL MC_4_R RN, PRKAG_3_	Fatty acid profile Meat tenderness Marbling Growth and profile IMF/marbling Meat quality/stress Growth and fatness Meat quality	[185] [184] [186] [187] [174] [188] [189] [190]
	AFABP/FABP_4_	IMF	[191]
	HFABP/FABP_3_	IMF	[192]
	CAST	Tenderness	[193]
	IGF_2_	Growth and fatness	[194]
Chicken	EX-FABP	Fatness	[195]
	L-FABP	Fatness	[196]

## References

[1] OECD Meat Consumption 2019. https://data.oecd.org/agroutput/meat-consumption.htm.

[2] Rowe J.B. (2010). The Australian sheep industry—Undergoing transformation. Anim. Prod. Sci..

[3] MLA Meat and Livestock Australia. Australia’s Sheep Meat Industry: Fresh Facts 2018. https://www.mla.com.au/globalassets/mla-corporate/prices--markets/documents/trends--analysis/fastfacts--maps/mla_sheep-fast-facts-2018.pdf.

[4] Milewski S. (2006). Health-promoting properties of sheep products. Med. Weter..

[5] Nuernberg K., Fisher A., Nuernberg G., Ender K., Dannenberger D. (2008). Meat quality and fatty acid composition of lipids in muscle and fatty tissue of Skudde lambs fed grass versus concentrate. Small Rum. Res..

[6] FAO Food and Agriculture Organization of the United Nations. Meat Quality 2014. http://www.fao.org/ag/againfo/themes/en/meat/quality_meat.

[7] MLA Meat and Livestock Australia. Eating Quality 2020. https://www.mla.com.au/research-and-development/feeding-finishing-nutrition/eating-quality.

[8] Cardoso C., Afonso C., Bandarra N.M. (2016). Seafood lipids and cardiovascular health. Nutrire.

[9] Scollan N.D., Dannenberger D., Nuernberg K., Richardson L., Mackintosh S., Hocquette J.F., Moloney A.P. (2014). Enhancing the nutritional and health value of beef lipids and their relationship with meat quality. Meat Sci..

[10] De Smet S., Vossen E. (2016). Meat: The balance between nutrition and health: A review. Meat Sci..

[11] Gu Z., Suburu J., Chen H., Chen Q.C. (2013). Mechanisms of omega-3 polyunsaturated fatty acids in prostate cancer prevention. Biomed. Res. Int..

[12] Calviello G., Serini S., Piccioni E. (2009). n-3 polyunsaturated fatty acids and the prevention of colorectal cancer: Molecular mechanisms involved. Curr. Med. Chem..

[13] Chi S.-C., Tuan H.-I., Kang Y.-N. (2019). Effects of polyunsaturated fatty acids on nonspecific typical dry eye disease: A systematic review and meta-analysis of randomized clinical trials. Nutrients.

[14] Zhang L., Liu H., Kuang L., Meng H., Zhou X. (2019). Omega-3 fatty acids for the treatment of depressive disorders in children and adolescents: A meta-analysis of randomized placebo-controlled trials. Child Adolesc. Psychiatry Ment. Health.

[15] Funaki M. (2009). Saturated fatty acids and insulin resistance. J. Med. Inv..

[16] Sripetchwandee J., Chattipakorn N., Chattipakorn S.C. (2018). Links between obesity-induced brain insulin resistance, brain mitochondrial dysfunction, and dementia. Front. Endocrinol..

[17] Janssen C.I.F., Kiliaan A.J. (2014). Long-chain polyunsaturated fatty acids (LCPUFA) from genesis to senescence: The influence of LCPUFA on neural development, aging and neurodegeneration. Prog. Lipid Res..

[18] Wood J.D., Enser M., Fisher A.V., Nute G.R., Sheard P.R., Richardson R.I., Hughes S.I., Whittington F.M. (2008). Fat deposition, fatty acid composition and meat quality: A review. Meat Sci..

[19] Scollan N.D., Hocquette J.F., Nuernberg K., Dannenberger D., Richardson I., Moloney A. (2006). Innovations in beef production systems that enhance the nutritional and health value of beef lipids and their relationship with meat quality. Meat Sci..

[20] Wood J.D., Richardson R.I., Nute G.R., Fisher A.V., Campo M.M., Kasapidou E., Sheard P.R., Enser M. (2004). Effects of fatty acids on meat quality: A review. Meat Sci..

[21] Webb E.C., O’Neill H.A. (2008). The animal fat paradox and meat quality. Meat Sci..

[22] Mwangi F.W., Charmley E., Gardiner C.P., Malau-Aduli B.S., Kinobe R.T., Malau-Aduli A.E.O. (2019). Diet and genetics influence beef cattle performance and meat quality characteristics. Foods.

[23] Ladeira M.M., Schoonmaker J.P., Gionbelli M.P., Dias J.C.O., Gionbelli T.R.S., Carvalho J.R.R., Teixeira P.D. (2016). Nutrigenomics and beef quality: A review about lipogenesis. Int. J. Mol. Sci..

[24] Calder P.C., Adolph M., Deutz N.E., Grau T., Innes J.K., Klek S., Lev S., Mayer K., Michael-Titus A.T., Pradelli L. (2018). Lipids in the intensive care unit: Recommendations from the ESPEN Expert Group. Clin. Nutr..

[25] Kenar J.A., Moser B.R., List G.R., Ahmad M.U. (2017). Naturally occurring fatty acids: Source, chemistry and uses. Fatty Acids: Chemistry, Synthesis and Applications.

[26] Patterson E., Wall R., Fitzgerald G.F., Ross R.P., Stanton C. (2012). Health implications of high dietary omega-6 polyunsaturated fatty acids. J. Nutr. Metab..

[27] Leyland B., Boussiba S., Khozin-Goldberg I. (2020). A review of diatom lipid droplets. Biology.

[28] Hayakawa K., Sakamoto T., Ishii A., Yamaji K., Uemoto Y., Sasago N., Kobayashi E., Kobayashi N., Matsuhashi T., Maruyama S. (2015). The g.841G>C SNP of *FASN* gene is associated with fatty acid composition in beef cattle. Anim. Sci. J..

[29] Melton S.L., Black J.M., Davis G.W., Backus W.R. (1982). Flavour and selected chemical components of ground beef from steers backgrounded on pasture and fed corn up to 140 days. J. Food Sci..

[30] Zhao Y., Wang C. (2018). Effect of ω-3 polyunsaturated fatty acid-supplemented parenteral nutrition on inflammatory and immune function in postoperative patients with gastrointestinal malignancy. A meta-analysis of randomized control trials in China. Medicine.

[31] Wall R., Ross R.P., Fitzgerald G.F., Stanton C. (2010). Fatty acids from fish: The anti-inflammatory potential of long-chain omega-3 fatty acids. Nutr. Rev..

[32] Insel P., Ross D., McMahon K., Bernstein M. (2018). Nutrition.

[33] Khan W.A., Chun-Mei H., Khan N., Iqbal A., Lyu S.W., Shah F. (2017). Bioengineered plants can be a useful source of omega-3 fatty acids. Biomed. Res. Int..

[34] Yehuda S., Rabinovitz S., Mostofsky D.I. (2005). Essential fatty acids and the brain: From infancy to aging. Neurobiol. Aging.

[35] Sampath H., Ntambi J.M. (2011). The role of fatty acid desaturases in epidermal metabolism. Dermato-endocrinology.

[36] Tao L., Sun T., Magnuson A.D., Qamar T.R., Xin Gen Lei X.G. (2018). Defatted microalgae-mediated enrichment of n–3 polyunsaturated fatty acids in chicken muscle is not affected by dietary selenium, vitamin E, or corn oil. J. Nutr..

[37] Calder P.C. (2016). Docosahexaenoic acid. Ann. Nutr. Metab..

[38] Calder P.C. (2014). Very long chain omega-3 (n-3) fatty acids and human health. Eur. J. Lipid Sci. Technol..

[39] Mayer K., Seeger W. (2008). Fish oil in critical illness. Curr. Opin. Clin. Nutr. Metab. Care.

[40] Tevar R., Jho D.H., Babcock T., Helton W.S., Espat N.J. (2002). Omega-3 fatty acid supplementation reduces tumor growth and vascular endothelial growth factor expression in a model of progressive non-metastasizing malignancy. JPEN J. Parenter. Enteral. Nutr..

[41] Calder P.C., Yaqoob P. (2009). Understanding omega-3 polyunsaturated fatty acids. Postgrad. Med..

[42] Weylandt K.H. (2016). Docosapentaenoic acid derived metabolites and mediators—The new world of lipid mediator medicine in a nutshell. Eur. J. Pharm..

[43] Kaur G., Cameron-Smith D., Garg M., Sinclair A.J. (2011). Docosapentaenoic acid (22:5n-3): A review of its biological effects. Prog. Lipid Res..

[44] Simopoulos A.P. (2016). An increase in the omega-6/omega-3 fatty acid ratio increases the risk for obesity. Nutrients.

[45] Benjamin E.J., Blaha M.J., Chiuve S.E., Cushman M., Muntner P. (2017). Heart disease and stroke statistics-2017 update: A report from the American Heart Association. Circulation.

[46] Siegel R.L., Miller K.D., Jemal A. (2017). Cancer statistics 2016. CA Cancer J. Clin..

[47] Nichols P.D., Kitessa S.M., Abeywardena M. (2014). Commentary on a trial comparing krill oil versus fish oil. Lipids Health Dis..

[48] Mozaffarian D., Lemaitre R.N., King I.B., Song X., Spiegelman D., Sacks F.M., Rimm E.B., Siscovick D.S. (2011). Circulating long-chain ω-3 fatty acids and incidence of congestive heart failure in older adults: The cardiovascular health study: A cohort study. Ann. Intern. Med..

[49] Cartwright I.J., Pockley G., Galloway J.H., Greaves M., Preston E. (1985). The effects of dietary omega-3 polyunsaturated fatty acids on erythrocyte membrane phospholipids, erythrocyte deformability and blood viscosity in healthy volunteers. Atherosclerosis.

[50] Watanabe Y., Tatsuno I. (2017). Omega-3 polyunsaturated fatty acids for cardiovascular diseases: Present, past and future. Expert Rev. Clin. Pharmacol..

[51] Simopoulos A.P. (2002). The importance of the ratio of omega-6/omega-3 essential fatty acids. Biomed. Pharmacother..

[52] Abeywardena M.Y., Head R.J. (2001). Long chain *n*−3 polyunsaturated fatty acids and blood vessel function. Cardiovasc. Res..

[53] Swanson D., Block R., Mousa S.A. (2012). Omega-3 fatty acids EPA and DHA: Health benefits throughout life. Adv. Nutr..

[54] Koletzko B., Lien E., Agostoni C., Bohles H., Campoy C., Cetin I., Decsi T., Dudenhausen J.W., Dupont C. (2008). The roles of long-chain polyunsaturated fatty acids in pregnancy, lactation and infancy: Review of current knowledge and consensus recommendations. J. Perinat. Med..

[55] Astorg P. (2004). Dietary n-6 and n-3 polyunsaturated fatty acids and prostate cancer risk: A review of epidemiological and experimental evidence. Cancer Causes Control.

[56] Leitzmann M., Stampfer M., Michaud D., Augustsson K., Colditz G., Willett W., Giovannucci E. (2004). Dietary intake of n-3 and n-6 fatty acids and the risk of prostate cancer. Am. J. Clin. Nutr..

[57] Welch A.A., Shakya-Shrestha S., Lentjes M.A.H., Wareham N.J., Khaw K.T. (2010). Dietary intake and status of n-3 polyunsaturated fatty acids in a population of fish-eating and non-fish-eating meat-eaters, vegetarians, and vegans and the precursor-product ratio of α-linolenic acid to long-chain n-3 polyunsaturated fatty acids: Results from the EPIC-Norfolk cohort. Am. J. Clin. Nutr..

[58] Gould J.F., Smithers L.G., Makrides M. (2013). The effect of maternal omega-3 (n-3) LCPUFA supplementation during pregnancy on early childhood cognitive and visual development: A systematic review and meta-analysis of randomized controlled trials. Am. J. Clin. Nutr..

[59] Calder P.C. (2017). New evidence that omega-3 fatty acids have a role in primary prevention of coronary heart disease. J. Public Health Emerg..

[60] Certik M., Shimizu S. (1999). Biosynthesis and regulation of microbial polyunsaturated fatty acid production. J. Biosci. Bioeng..

[61] Jacobs D.R., Ruzzin J., Lee D.H. (2014). Environmental pollutants: Downgrading the fish food stock affects chronic disease risk. J. Intern. Med..

[62] Orsavova J., Misurcova L., Ambrozova J.V., Vicha R., Mlcek J. (2015). Fatty acid composition of vegetable oils and its contribution to dietary energy intake and dependence of cardiovascular mortality on dietary intake of fatty acids. Int. J. Mol. Sci..

[63] Mori T.A. (2014). Omega-3 fatty acids and cardiovascular disease: Epidemiology and effects on cardiometabolic risk factors. Food Funct..

[64] Brenna J.T., Salem N., Sinclair A.J., Cunnane S.C. (2009). Alpha-linolenic acid supplementation and conversion to n-3 long-chain polyunsaturated fatty acids in humans. Prostaglandins Leukot. Essent. Fatty Acids.

[65] Kris-Etherton P.M., Taylor D.S., Yu-Poth S., Huth P., Moriarty K., Fishell V., Hargrove R.L., Zhao G.X., Etherton T.D. (2000). Polyunsaturated fatty acids in the food chain in the United States. Am. J. Clin. Nutr..

[66] Lum K.K., Kim J., Lei X.G. (2013). Dual potential of microalgae as a sustainable biofuel feedstock and animal feed. J. Anim. Sci. Biotechnol..

[67] Gregory M.K., Geier M.S., Gibson R.A., James M.J. (2013). Functional characterization of the chicken fatty acid elongases. J. Nutr..

[68] Yazdi P.G. (2013). A review of the biologic and pharmacologic role of docosapentaenoic acid n-3. F1000Research.

[69] Sun Q., Ma J., Campos H., Rexrode K.M., Albert C.M., Mozaffarian D., Hu F.B. (2008). Blood concentrations of individual long-chain n–3 fatty acids and risk of nonfatal myocardial infarction. Am. J. Clin. Nutr..

[70] Reinders I., Virtanen J.K., Brouwer I.A., Tuomainen T.P. (2002). Association of serum n-3 polyunsaturated fatty acids with C-reactive protein in men. Eur. J. Clin. Nutr..

[71] Rissanen T., Voutilainen S., Nyyssonen K., Lakka T.A., Salonen J.T. (2000). Fish oil–derived fatty acids, docosahexaenoic acid and docosapentaenoic acid, and the risk of acute coronary events: The Kuopio ischaemic heart disease risk factor study. Circulation.

[72] Aase K., Ernkvist M., Ebarasi L., Jakobsson L., Majumdar A., Yi C., Birot O., Ming Y., Kvanta A., Edholm D. (2007). Angiomotin regulates endothelial cell migration during embryonic angiogenesis. Genes Dev..

[73] Phang M., Garg M.L., Sinclair A.J. (2009). Inhibition of platelet aggregation by omega-3 polyunsaturated fatty acids is gender specific—Redefining platelet response to fish oils. Prostagland. Leukot. Essen. Fatty Acids..

[74] Augustsson K., Michaud D.S., Rimm E.B., Leitzman M.F., Stampfer M.J., Walter C.W., Giovannucci E. (2003). A prospective study of intake of fish and marine fatty acids and prostate cancer. Cancer Epidemiol. Biomark. Prev..

[75] Kanayasu-Toyoda T., Morita I., Murota S.I. (1996). Docosapentaenoic acid (22:5, n-3), an elongation metabolite of eicosapentaenoic acid (20:5, n-3), is a potent stimulator of endothelial cell migration on pretreatment in vitro. Prostagland. Leukot. Essen. Fatty Acids..

[76] Chen J., Jiang Y., Liang Y., Tian X., Peng C., Ma K.Y., Liu J., Huang Y., Chen Z.Y. (2012). DPA n-3, DPA n-6 and DHA improve lipoprotein profiles and aortic function in hamsters fed a high cholesterol diet. Atherosclerosis.

[77] Malau-Aduli A.E.O., Holman B.W.B., Khatib H. (2015). Molecular genetics-nutrition interactions in ruminant fatty acid metabolism and meat quality. Molecular and Quantitative Animal Genetics.

[78] Warren H.E., Scollan N.D., Enser M., Hughes S.I., Richardson R.I., Wood J.D. (2008). Effects of breed and a concentrate or grass silage diet on beef quality in cattle of 3 ages. I: Animal performance, carcass quality and muscle fatty acid composition. Meat Sci..

[79] National Health and Medical Research Council, Australian Government Department of Health and Ageing (2006). Nutrient Reference Values for Australia and New Zealand. Canberra: National Health and Medical Research Council. https://www.nhmrc.gov.au/sites/default/files/images/nutrient-refererence-dietary-intakes.pdf.

[80] Le H.V., Nguyen D.V., Nguyen Q.V., Malau-Aduli B.S., Nichols P.D., Malau-Aduli A.E.O. (2019). Fatty acid profiles of muscle, liver, heart and kidney of Australian prime lambs fed different polyunsaturated fatty acids enriched pellets in a feedlot system. Sci. Rep..

[81] Ponnampalam E.N., Bunter K.L., Pearce K.M., Mortimer S.I., Pethick D.W., Ball A.J., Hopkins D.L. (2014). Sources of variation of health claimable long chain omega-3 fatty acids in meat from Australian lamb slaughtered at similar weights. Meat Sci..

[82] Commission Regulation of European Union (2014). Statement on a conceptual framework for the risk assessment of certain food additives re-evaluated under Commission Regulation (EU) No 257/20102014. European Food Safety Authority Panel on Food additives and Nutrient Sources added to Food (ANS). EFSA J..

[83] World Health Organization (1990). Diet, Nutrition and the Prevention of Chronic Diseases.

[84] Simopoulos A.P. (2011). Evolutionary aspects of diet: The omega-6/omega-3 ratio and the brain. Mol. Neurobiol..

[85] Simopoulos A.P. (2008). The importance of the omega-6/omega-3 fatty acid ratio in cardiovascular disease and other chronic diseases. Expt. Biol. Med..

[86] Stark K.D., Van Elswyk M.E., Higgins M.R., Weatherford C.A., Salem N. (2016). Global survey of the omega-3 fatty acids, docosahexaenoic acid and eicosapentaenoic acid in the blood stream of healthy adults. Prog. Lipid Res..

[87] Corsinovi L., Biasi F., Poli G., Leonarduzzi G., Isaia G. (2011). Dietary lipids and their oxidized products in Alzheimer’s disease. Mol. Nutr. Food Res..

[88] Pighin D., Pazos A., Chamorro V., Paschetta F., Cunzolo S., Godoy F., Messina V., Pordomingo A., Grigioni G. A contribution of beef to human health: A review of the role of the animal production systems. Sci. World J..

[89] Henderson G., Cox F., Ganesh S., Jonker A., Young W., Global R.C.C., Janssen P.H. (2015). Rumen microbial community composition varies with diet and host, but a core microbiome is found across a wide geographical range. Sci. Rep..

[90] Morgavi D., Forano E., Martin C., Newbold C. (2010). Microbial ecosystem and methanogenesis in ruminants. Animal.

[91] Doreau M., Meynadier A., Fievez V., Ferlay A., Watson F.F., de Meester F. (2016). Ruminal Metabolism of Fatty Acids: Modulation of Polyunsaturated, Conjugated and Trans fatty Acids in Meat and Milk In Handbook of Lipids in Human Function: Fatty Acids.

[92] Demeyer D.I., Mertens B., De Smet S., Ulens M. (2016). Mechanisms linking colorectal cancer to the consumption of processed red meat: A Review. Crit. Rev. Food Sci. Nutr..

[93] Jenkins T.C., Wallace R.J., Moate P.J., Mosley E.E. (2008). Recent advances in biohydrogenation of unsaturated fatty acids within the rumen microbial ecosystem. J. Anim. Sci..

[94] Beam T.M., Jenkins T.C., Moate P.J., Kohn K.A., Palmquist D.L. (2000). Effects of amount and source of fat on the rates of lipolysis and biohydrogenation of fatty acids in ruminal contents. J. Dairy Sci..

[95] Buccioni A., Decandia M., Minieri S., Molle G., Cabiddu A. (2012). Lipid metabolism in the rumen: New insights on lipolysis and biohydrogenation with an emphasis on the role of endogenous plant factors. Anim. Feed Sci. Tech..

[96] Van Nevel C.J., Demeyer D.I. (1996). Influence of pH on lipolysis and biohydrogenation of soybean oil by rumen contents in vitro. Reprod. Nutr. Dev..

[97] Dewanckele L., Vlaeminck B., Hernandez-Sanabria E., Ruiz-Gonzalez A., Dubruyne S., Jeyanathan J., Frievez V. (2018). Rumen biohydrogenation and microbial community changes upon early life supplementation of 22:6*n*-3 enriched microalgae to goats. Front. Microbiol..

[98] Edwards H.D., Shelver W.L., Choi S., Nisbet D.J., Krueger N.A., Anderson R.C., Smith S.B. (2017). Immunogenic inhibition of prominent ruminal bacteria as a means to reduce lipolysis and biohydrogenation activity in vitro. Food Chem..

[99] Palmquist D.L., Lock A.L., Shingfield K.J., Bauman D.E. (2005). Biosynthesis of conjugated linoleic acid in ruminants and humans. Adv. Food Nutr. Res..

[100] Li D., Wang J.Q., Bu D.P. (2012). Ruminal microbe of hydrogenation of trans- vaccenic acid to stearic acid in vitro. BMC Res. Notes.

[101] Ferlay A., Bernard L., Meynadier A., Malpuech-Brugère C. (2017). Production of trans and conjugated fatty acids in dairy ruminants and their putative effects on human health: A review. Biochimie.

[102] Bickerstaffe D., Noakes D.E., Annison E.F. (1972). Quantitative aspects of fatty acid biohydrogenation, absorption and transfer into milk fat in the lactating goat, with special reference to the cis- and trans-isomers of octadecenoate and linoleate. Biochem. J..

[103] Griinari J.M., Bauman D.E. (1999). Biosynthesis of conjugated linoleic acid and its incorporation into meat and milk in ruminants. Advances in Conjugated Linoleic Acid Research.

[104] Demeyer D.I., Jouruay J.-P. (1991). Quantitative aspects of microbial metabolism in the rumen and hindgut. Rumen Microbial Metabolism and Ruminant Digestion, Institut Nationale de la Recherche Agronomique.

[105] Moss A.R., Jouany J.-P., Newbold J. (2000). Methane production by ruminants: Its contribution to global warming. Ann. Zootech..

[106] Ríos-Covián D., Ruas-Madiedo P., Margolles A., Gueimonde M., de Los Reyes-Gavilán C.G., Salazar N. (2016). Intestinal short chain fatty acids and their link with diet and human health. Front. Microbiol..

[107] Fruet A.P.B., Stefanello F.S., Trombetta F., De Souza A.N.M., Junior A.G.R., Tonetto C.J., Flores J.L.C., Scheibler R.B., Bianchi R.M., Pacheco P.S. (2019). Growth performance and carcass traits of steers finished on three different systems including legume–grass pasture and grain diets. Animal.

[108] Redfearn D.D., Venuto B.C., Pitman W.D., Alison M.W., Ward J.D. (2002). Cultivar and environmental effects on annual ryegrass forage yield, yield distribution, and nutritive value. Crop Sci..

[109] Buxton D., Hornstein J.S., Wedin W.F., Marten G.C. (1985). Forage quality in stratified canopies of alfalfa, birdsfoot trefoil and red clover. Crop Sci..

[110] Sleugh B., Moore K.J., George J.R., Brummer E.C. (2000). Binary legume-grass mixtures improve forage yield, quality, and seasonal distribution. Agron. J..

[111] Fruet A.P.B., Stefanello F.S., Júnior A.G.R., De Souza A.N.M., Tonetto C.J., Nörnberg J.L. (2016). Whole grains in the finishing of culled ewes in pasture or feedlot: Performance, carcass characteristics and meat quality. Meat Sci..

[112] Aldai N., Dugan M.E.R., Kramer J.K.G., Martinez A., Lopez-Campos O., Mantecon A.R., Osoro K. (2011). Length of concentrate finishing affects the fatty acid composition of grass-fed and genetically lean beef: An emphasis on trans-18:1 and conjugated linoleic acid profiles. Animal.

[113] Demirel G., Ozpinar H., Nazli B., Keser O. (2006). Fatty acids of lamb meat from two breeds fed different forage: Concentrate ratio. Meat Sci..

[114] Roberts S.D., Kerth C.R., Branden K., Rankins D.L., Kriese-Anderson L.A., Prevatt J.W. (2009). Finishing steers on winter annual ryegrass (*Lolium multiflorum* Lam.) with varied levels of corn supplementation I: Effects on animal performance, carcass traits, and forage quality. J. Anim. Sci..

[115] Duckett S.K., Neel J.P.S., Lewis R.M., Fontenot J.P., Clapham W.N. (2013). Effects of forage species or concentrate finishing on animal performance, carcass and meat quality. J. Anim. Sci..

[116] Realini C., Duckett S.K., Brito G., Dalla Rizza M., de Mattos D. (2004). Effect of pasture vs. concentrate feeding with or without antioxidants on carcass characteristics, fatty acid composition, and quality of Uruguayan beef. Meat Sci..

[117] Raes K., De Smet S., Demeyer D. (2004). Effect of dietary fatty acids on incorporation of long chain polyunsaturated fatty acids and conjugated linoleic acid in lamb, beef and pork meat: A review. Anim. Feed Sci. Tech..

[118] Arelovich H.M., Marinissen J., Gardner B.A., Martinez M.F., Bravo R.D. (2017). Effects of oats grain supplements on performance, rumen parameters and composition of beef from cattle grazing oats pasture. Anim. Prod. Sci..

[119] Rovadoscki G.A., Pertile S.F.N., Alvarenga A.B., Cesar A.S.M., Pértille F., Petrini J., Franzo V., Soares W.V.B., Morota G., Spangler M.L. (2018). Estimates of genomic heritability and genome-wide association study for fatty acids profile in Santa Inês sheep. BMC Genom..

[120] Tiezzi F., Parker-Gaddis K.L., Cole J.B., Clay J.S., Maltecca C. (2015). A genome-wide association study for clinical mastitis in first parity US Holstein cows using single-step approach and genomic matrix re-weighting procedure. PLoS ONE.

[121] Goddard M.E., Hayes B.J. (2007). Genomic selection. J. Anim. Breed. Genet..

[122] Bolormaa S., Pryce J.E., Kemper K., Savin K., Hayes B.J., Barendse W., Zhang Y., Reich C.M., Mason B.A., Bunch R.J. (2013). Accuracy of prediction of genomic breeding values for residual feed intake and carcass and meat quality traits in Bos taurus, Bos indicus, and composite beef cattle. J. Anim. Sci..

[123] Van Raden P.M. (2008). Efficient methods to compute genomic predictions. J. Dairy Sci..

[124] Meuwissen T.H., Hayes B.J., Goddard M.E. (2001). Prediction of total genetic value using genome-wide dense marker maps. Genetics.

[125] Casas E., White S.N., Wheeler T.L., Shackelford S.D., Koohmaraie M., Riley D.G., Chase C.C., Johnson D.D., Smith T.P.L. (2006). Effects of calpastatin and mu-calpain markers in beef cattle on tenderness traits. J. Anim. Sci..

[126] Hayes B.J., Bowman P.J., Chamberlain A.C., Goddard M.E. (2009). Genomic selection in dairy cattle: Progress and challenges. J. Dairy Sci..

[127] Maharani D., Jung Y., Jung W.Y., Jo C., Ryoo S.H., Lee S.H., Yeon S.H., Lee J.H. (2012). Association of five candidate genes with fatty acid composition in Korean cattle. Mol. Biol. Rep..

[128] Bhuiyan M.S.A., Yu S.L., Jeon J.T., Yoon D., Cho Y.M., Park E.W., Kim N.K., Kim K.S., Lee J.H. (2009). DNA polymorphisms in SREBF1 and FASN genes affect fatty acid composition in Korean cattle (Hanwoo). Asian-Aust. J. Anim. Sci..

[129] Esteves C., Livramento K.G., Paiva L.V., Peconick A.P., Garcia I.F.F., Garbossa C.A.P., Faria P.B. (2019). The polymorphisms of genes associated with the profile of fatty acids of sheep. Arq. Bras. Med. Vet. Zootec..

[130] Gu M., Cosenza G., Iannaccone M., Macciotta N.P.P., Guo Y., Di Stasio L., Pauciullo A. (2019). The single nucleotide polymorphism g.133A>C in the stearoyl CoA desaturase gene (SCD) promoter affects gene expression and quali-quantitative properties of river buffalo milk. J. Dairy Sci..

[131] Paton C.M., Ntambi J.M. (2009). Biochemical and physiological function of stearoyl-CoA desaturase. Am. J. Physiol. Endocrinol. Metab..

[132] Mannen H. (2011). Identification and utilization of genes associated with beef qualities. Anim. Sci. J..

[133] Milanesi E., Nicoloso L., Crepaldi P. (2008). Stearoyl CoA desaturase (SCD) gene polymorphisms in Italian cattle breeds. J. Anim. Breed. Genet..

[134] Lengi A.J., Corl B.A. (2007). Identification and characterization of a novel bovine stearoyl-coA desaturase isoform with homology to human SCD5. Lipids.

[135] Kim Y.C., Ntambi J.M. (1999). Regulation of stearoyl-CoA desaturase genes: Role in cellular metabolism and preadipocyte differentiation. Biochem. Biophys. Res. Commun..

[136] Smith S.B., Gill C.A., Lunt D.K., Brooks M.A. (2009). Regulation of fat and fatty acid composition in beef cattle. Asian-Aust. J. Anim. Sci..

[137] Bauman D.E., Mather I.H., Wall R.J., Lock A.L. (2006). Major advances associated with the biosynthesis of milk. J. Dairy Sci..

[138] Guillou H., Zadravec D., Martin P.G.P., Jacobsson A. (2010). The key roles of elongases and desaturases in mammalian fatty acid metabolism: Insights from transgenic mice. Prog. Lipid Res..

[139] Bhattacharya A., Banu J., Rahman M., Causey J., Fernandes G. (2006). Biological effects of conjugated linoleic acids in health and disease. J. Nutr. Biochem..

[140] Reynolds C.M., Roche H.M. (2010). Conjugated linoleic acid and inflammatory cell signaling. Prost. Leukot. Essent. Fatty Acids.

[141] Calvo J.H., González-Calvo L., Dervishi E., Blanco M., Iguácel L.P., Sarto P., Pérez-Campo F.M., Serrano M., Bolado-Carrancio A., Rodríguez-Rey J.C. (2019). A functional variant in the stearoyl-CoA desaturase (SCD) gene promoter affects gene expression in ovine muscle. Livest. Sci..

[142] Carta A., Casu S., Usai M., Addis M., Fiori M., Fraghi A., Miari S., Mura L., Piredda G., Schibler L. (2008). Investigating the genetic component of fatty acid content in sheep milk. Small Rum. Res..

[143] Armstrong E., Ciappesoni G., Iriarte W., Da Silva C., Macedo F., Navajas E.A., Brito G., San Julián R., Gimeno D., Postiglioni A. (2018). Novel genetic polymorphisms associated with carcass traits in grazing Texel sheep. Meat Sci..

[144] García-Fernández M., Gutiérrez-Gil B., García-Gámez E., Arranz J.J. (2009). Genetic variability of the stearoyl-CoA desaturase gene in sheep. Mol. Cell. Probes.

[145] Miyazaki M., Ntambi J.M. (2003). Role of stearoyl-coenzyme A desaturase in lipid metabolism. Prostaglandins Leukot. Essent. Fatty Acids.

[146] Dervishi E., Serrano C., Joy M., Serrano M., Rodellar C., Calvo J.H. (2010). Effect of the feeding system on the fatty acid composition, expression of the Δ9 -desaturase, Peroxisome Proliferator-Activated Receptor Alpha, Gamma, and Sterol Regulatory Element Binding Protein 1 genes in the semitendinous muscle of light lambs of the Rasa Aragonesa breed. BMC Vet. Res..

[147] Roy R., Gautier M., Hayes H., Laurent P., Osta R. (2001). Assignment of the fatty acid synthase (FASN) gene to bovine chromosome 19 (19q22) by in situ hybridization and confirmation by somatic cell hybrid mapping. Cytogenet. Cell. Genet..

[148] Chirala S.S., Chang H., Matzuk M., Abu-Elheiga L., Mao J., Mahson K., Finegold M., Wakil S.J. (2003). Fatty acid synthesis is essential in embryonic development: Fatty acid synthase null mutants and most of the heterozygotes die in utero. Proc. Natl. Acad. Sci. USA.

[149] Zhang S., Knight T.J., Reecy J.M., Beitz D.C. (2008). DNA polymorphisms in bovine fatty acid synthase are associated with beef fatty acid composition. Anim. Genet..

[150] Morris C.A., Cullen N.G., Glass B.G., Hyndman D.L., Manley T.R., Hickey S.M., McEwan J.S., Pitchford W.S., Bottema C.D.K., Lee M.A.H. (2007). Fatty acid synthase effects on bovine adipose fat and milk fat. Mamm. Genom..

[151] Roy R., Ordovas L., Zaragoza P., Romero A., Moreno C., Altarriba J., Rodellar C. (2006). Association of polymorphisms in the bovine FASN gene with milk fat content. Anim. Genet..

[152] Mohammed M.E.A., Abeer A., Elsamani F., Elsheikh O.M., Hodow A., Haji O.K. (2013). Simulation of the fatty acid synthase complex mechanism of action. Chem. Bulg. J. Sci. Edu..

[153] Chakravarty B., Gu Z., Chirala S.S., Wakil S.J., Quiocho F.A. (2004). Human fatty acid synthase: Structure and substrate selectivity of the thioesterase domain. Proc. Natl. Acad. Sci. USA.

[154] Oh D.-Y., Lee Y.-S., La B.-M., Yeo J.S., Chung E., Kim Y., Lee C. (2012). Fatty acid composition of beef is associated with exonic nucleotide variants of the gene encoding FASN. Mol. Biol. Rep..

[155] Sanz A., Serrano C., Ranera B., Dervishi E., Zaragoza P., Calvo J.H., Rodellar C. (2015). Novel polymorphisms in the 5′UTR of FASN, GPAM, MC4R and PLIN1 ovine candidate genes: Relationship with gene expression and diet. Small Rum. Res..

[156] Wang B., Yang L., Luo Y., Su R., Su L., Zhao L., Jin Y.E. (2018). Effects of feeding regimens on meat quality, fatty acid composition and metabolism as related to gene expression in Chinese Sunit sheep. Small Rum. Res..

[157] Ekegbu U.J., Burrows L., Amirpour-Hamed N., Zhou H., Hicford J.G.H. (2019). Gene polymorphisms in PROP1 associated with growth traits in sheep. Gene.

[158] Kucharski M., Kaczor U. (2017). Fatty Acid binding protein 4 (FABP4) and the body lipid balance. Folia Biologica.

[159] Latruffe N., Vamecq J. (1997). Peroxisome proliferators and peroxisome proliferator activated receptors (PPAR) as regulators of lipid metabolism. Biochimie.

[160] Li L., Jiang J., Wang L., Zhong T., Chen B., Zhan S., Zhang H., Du L. (2013). Expression patterns of peroxisome proliferator activated receptor gamma 1 versus gamma 2 and their association with intramuscular fat in goat tissues. Gene.

[161] Yan W., Zhou H., Luo Y.Z., Hu J., Hickford J.G. (2012). Allelic variation in ovine fatty acid-binding protein (FABP4) gene. Mol. Biol. Rep..

[162] Yan W., Zhou H., Hu J., Luo Y., Hickford J.G.H. (2018). Variation in the FABP4 gene affects carcass and growth traits in sheep. Meat Sci..

[163] Dervishi E., Serrano E., Joy M., Serrano M., Rodellar C., Calvo J.H. (2011). The effect of feeding system in the expression of genes related with fat metabolism in semitendinous muscle in sheep. Meat Sci..

[164] Barendse W., Bunch R.J., Thomas M.B., Harrison B.E. (2009). A splice site single nucleotide polymorphism of the fatty acid binding protein 4 gene appears to be associated with intramuscular fat deposition in longissimus muscle in Australian cattle. Anim. Genet..

[165] Hoashi S., Hinenoya T., Tanaka A., Ohsaki H., Sasazaki S., Taniguchi M., Oyama K., Mukai F., Mannen H. (2008). Association between fatty acid compositions and genotypes of FABP4 and LXR-alpha in Japanese Black cattle. BMC Genet..

[166] Ardicli S., Samli H., Alpay F., Dincel D., Soyudal B., Balci F. (2017). Association of single nucleotide polymorphisms in the FABP4 gene with carcass characteristics and meat quality in Holstein bulls. Ann. Anim. Sci..

[167] Dujková R., Ranganathan Y., Dufek A., Macák J., Bezdíˇcek J. (2015). Polymorphic effects of FABP4 and SCD genes on intramuscular fatty acid profiles in longissimus muscle from two cattle breeds. Acta Vet. BRNO.

[168] Cho S., Park T.S., Yoon D.H., Cheong H.S., Namgoong S., Park B.L., Lee H.W., Han C.S., Kim E.M., Cheong I.C. (2008). Identification of genetic polymorphisms in FABP3 and FABP4 and putative association with back fat thickness in Korean native cattle. BMB Rep..

[169] Bahnamiri H.Z., Zali A., Ganjkhanlou M., Sadaghi M., Sharbabak H.M. (2018). Regulation of lipid metabolism in adipose depots of fat-tailed and thin-tailed lambs during negative and positive energy balances. Gene.

[170] Backhtiarizadeh M.R., Moradi-Shahrbabak M., Ebrahimie E. (2013). Underlying functional genomics of fat deposition in adipose tissue. Gene.

[171] Storch J., Corsico B. (2008). The emerging functions and mechanisms of mammalian Fatty Acid-Binding Proteins. Ann. Rev. Nutr..

[172] Rempel L.A., Casas E., Shackelford S.D., Wheeler T.L. (2012). Relationship of polymorphisms within metabolic genes and carcass traits in crossbred beef cattle. J. Anim. Sci..

[173] Shin S.C., Chung E.R. (2006). Association of SNP marker in the leptin gene with carcass and meat quality traits in Korean cattle. Asian-Aust. J. Anim. Sci..

[174] Thaller G., Kühn C., Winter A., Ewald G., Bellmann O., Wegner J., Zühlke H., Fries R. (2003). DGAT1, a new positional and functional candidate gene for intramuscular fat deposition in cattle. Anim. Genet..

[175] Barendse W., Bunch R.J., Harrison B.E., Thomas M.B. (2006). The growth hormone 1 GH1: C.457C & gtG mutation is associated with intramuscular and rump fat distribution in a large sample of Australian feedlot cattle. Anim. Genet..

[176] Gao Y., Zhang R., Hu X., Li N. (2007). Application of genomic technologies to the improvement of meat quality of farm animals. Meat Sci..

[177] Greguła-Kania M., Gruszecki T.M., Junkuszew A., Juszczuk-Kubiak E., Florek M. (2019). Association of CAST gene polymorphism with carcass value and meat quality in two synthetic lines of sheep. Meat Sci..

[178] Grochowska E., Borys B., Lisiak D., Mroczkowski S. (2019). Genotypic and allelic effects of the myostatin gene (MSTN) on carcass, meat quality, and biometric traits in Colored Polish Merino sheep. Meat Sci..

[179] Fan Y., Ren C., Meng F., Deng K., Zhang G., Wang F. (2019). Effects of algae supplementation in high-energy dietary on fatty acid composition and the expression of genes involved in lipid metabolism in Hu sheep managed under intensive finishing system. Meat Sci..

[180] Wang J., Zhou H., Forrest R.H.J., Hu J., Li S., Luo Y., Hickford J.G.H. (2017). Variation in the ovine MYF5 gene and its effect on carcass lean meat yield in New Zealand Romney sheep. Meat Sci..

[181] Freking B.A., Murphy S.K., Wylie A.A., Rhodes S.J., Keele J.W., Leymaster K.A., Jirtle R.L., Smith T.P. (2002). Identification of the single base change causing the callipyge muscle hypertrophy phenotype, the only known example of polar overdominance in mammals. Genome Res..

[182] Clop A., Marcq F., Takeda H., Pirottin D., Tordoir X., Bibe B., Bouix J., Caiment F., Elsen J.M., Eychenne F. (2006). A mutation creating a potential illegitimate microRNA target site in the myostatin gene affects muscularity in sheep. Nat. Genet..

[183] Malau-Aduli A.E.O., Bignell C.W., McCulloch R., Kijas J.W., Nichols P.D., De Smet S. Genetic association of delta-six fatty acid desaturase single nucleotide polymorphic molecular marker and muscle long chain omega-3 fatty acids in Australian lamb. Global Challenges to Production, Processing and Consumption of Meat, Proceedings of the 57th International Congress of Meat Science and Technology.

[184] Knight M.I., Daetwyler H.D., Hayes B.J., Hayden M.J., Ball A.J., Pethick D.W., McDonagh M.B. (2014). An independent validation association study of carcass quality, shear force, intramuscular fat percentage and omega-3 polyunsaturated fatty acid content with gene markers in Australian lamb. Meat Sci..

[185] Da Costa A.S.H., Pires V.M.R., Fontes C.M.D.A., Prates J.A.M. (2013). Expression of genes controlling fat deposition in two genetically diverse beef cattle breeds fed high or low silage diets. BMC Vet. Res..

[186] Anton I., Kovacs K., Hollo G., Farkas V., Lehel L., Hajda Z., Zsolnai A. (2011). Effect of leptin, DGAT1 and TG gene polymorphisms on the intramuscular fat of Angus cattle in Hungary. Livest. Sci..

[187] Mullen A.M., Stapleton P.C., Corcoran D., Hamill R.M., White A. (2006). Understanding meat quality through the application of genomic and proteomic approaches. Meat Sci..

[188] Fujii J., Otsu K., Zorzato F., de Leon S., Khanna V.K., Weiler J.E., O’Brien P.J., MacLennan D.H. (1991). Identification of a mutation in porcine ryanodine receptor associated with malignant hyperthermia. Science.

[189] Kim K.S., Larsen N., Short T., Plastow G., Rothschild M.F. (2000). A missense variant of the porcine melanocortin-4 receptor (MC4R) gene is associated with fatness, growth, and feed intake traits. Mamm. Genome.

[190] Milan D., Jeon J.T., Looft C., Amarger V., Robic A., Thelander M., Rogel-Gaillard C., Paul S., Iannuccelli N., Rask L. (2000). A mutation in PRKAG3 associated with excess glycogen content in pig skeletal muscle. Science.

[191] Gerbens F., Jansen A., van Erp A.J., Harders F., Meuwissen T.H., Rettenberger G., Veerkamp J.H., Te Pas M.F. (1998). The adipocyte fatty acid-binding protein locus: Characterization and association with intramuscular fat content in pigs. Mamm. Genome.

[192] Gerbens F., van Erp A.J., Harders F.L., Verburg F.J., Meuwissen T.H., Veerkamp J.H., Te Pas M.F.W. (1999). Effect of genetic variants of the heart fatty acid-binding protein gene on intramuscular fat and performance traits in pigs. J. Anim. Sci..

[193] Ciobanu D.C., Bastiaansen J.W., Lonergan S.M., Thomsen H., Dekkers J.C., Plastow G.S., Rothschild M.F. (2004). New alleles in calpastatin gene are associated with meat quality traits in pigs. J. Anim. Sci..

[194] Van Laere A.S., Nguyen M., Braunschweig M., Nezer C., Collette C., Moreau L., Archibald A.L., Haley C.S., Buys N., Tally M. (2003). A regulatory mutation in IGF2 causes a major QTL effect on muscle growth in the pig. Nature.

[195] Wang Q.G., Li N., Deng X.M., Lian Z.X., Li H., Wu C.X. (2001). Single nucleotide polymorphism analysis on chicken extracellular fatty acid binding protein gene and its associations with fattiness trait. Sci. China.

[196] Wang Q., Li H., Li N., Leng L., Wang Y. (2006). Tissue expression and association with fatness traits of liver fatty acid-binding protein gene in chicken. Poultry Sci..

[197] Pannier L., Gardner G.E., O’Reilly R.A., Pethick D.W. (2018). Factors affecting lamb eating quality and the potential for their integration into an MSA sheepmeat grading model. Meat Sci..

[198] Lambe N.R., McLean K.A., Gordon J., Evans D., Clelland N., Bunger L. (2017). Prediction of intramuscular fat content using CT scanning of packaged lamb cuts and relationships with meat eating quality. Meat Sci..

[199] Flakemore A., McEvoy P.D., Balogun R.O., Malau-Aduli B.S., Nichols P.D., Malau-Aduli A.E.O. (2014). Degummed crude canola oil supplementation affects fat depot melting points in purebred and first-cross Merino sheep. Anim. Vet. Sci..

[200] Calnan H.B., Jacob R.H., Pethick D.W., Gardner G.E. (2017). Selection for intramuscular fat and lean meat yield will improve the bloomed colour of Australian lamb loin meat. Meat Sci..

[201] Hocquette J.F., Richardson R.I., Prache S., Medale F., Duffy G., Scollan N.D. (2005). The future trends for research on quality and safety of animal products. Ital. J. Anim. Sci..

[202] Indurain G., Carr T.R., Goñi M.V., Insausti K., Beriain M.J. (2009). The relationship of carcass measurements to carcass composition and intramuscular fat in Spanish beef. Meat Sci..

[203] Joo S.T., Kim G.D., Hwang Y.H., Ryu Y.C. (2013). Control of fresh meat quality through manipulation of muscle fiber characteristics. Meat Sci..

[204] Park G.B., Moon S.S., Ko Y.D., Ha J.K., Lee J.G., Chang H.H., Joo S.T. (2002). Influence of slaughter weight and sex on yield and quality grades of Hanwoo (Korean native cattle) carcasses. J. Anim. Sci..

[205] Holman B.W.B., Malau-Aduli A.E.O. (2013). Spirulina as a livestock supplement and animal feed. J. Anim. Physiol. Anim. Nutr..

[206] Smith S.B., Kawachi H., Choi C.B., Choi C.W., Wu G., Sawyer J.E. (2009). Cellular regulation of bovine intramuscular adipose tissue development and composition. J. Anim. Sci..

[207] Hausman G.J., Dodson M.V., Ajuwon K., Azain M., Barnes K.M., Guan L.L., Jiang Z., Poulos S.P., Sainz E.D., Smith S. (2009). Board-Invited Review: The biology and regulation of preadipocyte and adipocytes in meat animals, *J*. Anim. Sci..

[208] Hocquette J.F., Mainsant P., Daudin J.D., Cassar-Malek I., Rémond D., Doreau M., Sans P., Bauchart D., Agabriel J., Verbecke W. (2013). Will meat be produced in vitro in the future?. INRA Prod. Anim..

[209] Wyness L. (2013). Nutritional aspects of red meat in the diet. Nutrition and Climate Change: Major Issues Confronting the Meat Industry, Wood, J.D., Rowlings, C., Eds..

[210] Wall K.R., Kerth C.R., Miller R.K., Alvarado C. (2019). Grilling temperature effects on tenderness, juiciness, flavor and volatile aroma compounds of aged ribeye, strip loin, and top sirloin steaks. Meat Sci..

[211] Koohmaraie R.A., Whipple G., Crouse L. (1990). Acceleration of postmortem tenderization in lamb and Brahaman-cross beef carcasses through infusion of calcium chloride. J. Anim Sci..

[212] Koohmaraie M., Kent M.P., Shackelford S.D., Veiseth E., Wheeler T.M. (2002). Meat tenderness and muscle growth: Is there any relationship?. Meat Sci..

[213] Cohen-Zinder M., Orlov A., Trofimyuk O., Agmon R., Kabiya R., Shor-Shimoni E., Wagner E.K., Hussey K., Leibovich H., Miron J. (2017). Dietary supplementation of Moringa oleifera silage increases meat tenderness of Assaf lambs. Small Rum. Res..

[214] Muchenje V., Dzama K., Chimonyo M., Strydom P.E., Raats J.G. (2009). Relationship between pre-slaughter responsiveness and beef quality in three cattle breeds. Meat Sci..

[215] Razminowicz R.H., Kreuzer M., Scheeder M.R.L. (2006). Quality of retail beef from two grass-based production systems in comparison with conventional beef. Meat Sci..

[216] Perlo F., Bonato P., Teira G., Tisocco O., Vicentin J., Pueyo J., Mansilla A. (2008). Meat quality of lambs produced in the Mesopotamia region of Argentina finished on different diets. Meat Sci..

[217] Sañudo C., Alfonso M., Sanchez A., Berge P., Dransfield E., Zygoyiannis D., Stamataris C., Thorkelsson G., Valdimarsdottir T., Piasentier E. (2003). Meat texture of lambs from different European production systems. Aust. J. Agric. Res..

[218] De Brito G.F., Ponnampalam E.R., Hopkins D.L. (2017). The effect of extensive feeding systems on growth rate, carcass traits, and meat quality of finishing lambs. Compr. Rev. Food Sci. Food Saf..

[219] Montossi F., Font-i-Furnols M., del Campo M., San Julián R., Brito G., Sañudo C. (2013). Sustainable sheep production and consumer preference trends: Compatibilities, contradictions, and unresolved dilemmas. Meat Sci..

[220] Yang C., Liu J., Wu X., Bao P., Long R., Guo X., Ding X., Yan P. (2017). The response of gene expression associated with lipid metabolism, fat deposition and fatty acid profile in the *longissimus dorsi* muscle of Gannan yaks to different energy levels of diets. PLoS ONE.

[221] Jurie C., Cassarmalek I., Bonnet M., Leroux C., Bauchart D., Boulesteix P., Pethick D.W., Hocquette J.F. (2007). Adipocyte fatty acid-binding protein and mitochondrial enzyme activities in muscles as relevant indicators of marbling in cattle. J. Anim Sci..

[222] Oliveira D.M., Chalfun-Junior A., Chizzotti M.L., Barreto H.G., Coelho T.C., Paiva L.V., Coelho C.P., Teixeira P.D., Schoonmaker J.P., Ladeira M.M. (2014). Expression of genes involved in lipid metabolism in the muscle of beef cattle fed soybean or rumen-protected fat, with or without monensin supplementation. J. Anim Sci..

[223] Lusk J.L., Fox J.A., Schroeder T.C., Mintert J., Koohmaraie M. (2001). In-store valuation of steak tenderness. Am. J. Agric. Econ..

[224] Elmasry G., Barbin D.F., Sun D., Allen P. (2002). Meat quality evaluation by hyperspectral imaging technique: An overview. Crit. Rev. Food Sci. Nutr..

[225] Ali M.S., Yang H.S., Jeong J.Y., Moon S.H., Hwang Y.H., Park G.B., Joo S.T. (2008). Effects of chilling temperature of carcass on breast meat quality of duck. Poult. Sci..

[226] Starkey C.P., Geesink G.H., Collins D., Oddy V.H., Hopkins D.L. (2016). Do sarcomere length, collagen content, pH, intramuscular fat and desmin degradation explain variation in the tenderness of three ovine muscles?. Meat Sci..

[227] Rhee M.S., Wheeler T.L., Shackelford S.D., Koohmaraie M. (2004). Variation in palatability and biochemical traits within and among eleven beef muscles. J. Anim. Sci..

[228] Guzek D., Głąska D., Pogorzelski G., Kozań K., Pietras J., Konarska M., Sakowska A., Głąski K., Pogorzelska E., Barszczewski J. (2013). Variation of meat quality parameters due to conformation and fat class in Limousin bulls slaughtered at 25 to 27 months of age. Asian-Aust. J. Anim. Sci..

[229] Arshad M.S., Sohaib M., Ahmad R.S., Nadeem M.T., Imran A., Arshad M.U., Kwon J.H., Amjad Z. (2018). Ruminant meat flavour influenced by different factors with special reference to fatty acids. Lipids Hlth. Dis..

[230] Khan M.I., Jang S., Nam K.C., Jo C. (2016). Postmortem aging of beef with a special reference to dry aging. Korean J. Food Sci. Anim. Resour..

[231] Calkins C.R., Hodgen J.M. (2007). A fresh look at meat flavour. Meat Sci..

[232] Mottram D.S. (1998). Flavour formation in meat and meat products: A review. Food Chem..

[233] Mottram D.S., Piggott J.R., Paterson A. (1994). Meat flavour. Understanding Natural Flavors.

[234] Jamora J.J., Rhee K.S., Xiong Y.L., Chi-Tang H., Shahidi F. (1999). Flavour of lamb and mutton. Quality Attributes of Muscle Foods.

[235] Muir P.D., Deaker J.M., Bown M.D. (1998). Effects of forage- and grain-based feeding systems on beef quality: A review. New Zeal. J. Agr. Res..

[236] McMillin K.W., Hoffman L.C., Kerry J. (2009). Improving the quality of meat from ratites. Improving the Sensory and Nutritional Quality of Fresh Meat.

[237] de Lima D.M., de Carvalho F.F.R., da Silva F.J.S., Rangel A.H., Novaes L.P., Difante G.D.S. (2016). Intrinsic factors affecting sheep meat quality: A review. Rev. Colomb. Cienc. Pecu..

[238] Cloete J.J.E., Hoffman L.C., Cloete S.W.P. (2012). A comparison between slaughter traits and meat quality of various sheep breeds: Wool, dual-purpose and mutton. Meat Sci..

[239] Hocquette J.F., Gondret F., Baéza E., Medale F., Jurie C., Pethick D.W. (2010). Intramuscular fat content in meat-producing animals: Development, genetic and nutritional control, and identification of putative markers. Animal.

[240] Jeremiah L.E., Gibson L.L., Aalhus J.L., Dugan M.E.R. (2003). Assessment of palatability attributes of the major beef muscles. Meat Sci..

[241] Aaslyng M.D., Oksama M., Olsen E.V., Bejerholm C., Baltzer M., Andersen G., Bredie W.L.P., Byrne D.V., Gabrielsen G. (2007). The impact of sensory quality of pork on consumer preference. Meat Sci..

[242] Cho S.H., Kim J., Park B.Y., Seong P.N., Kang G.H., Kim J.H., Jung S.G., Im S.K., Kim D.H. (2010). Assessment of meat quality properties and development of a palatability prediction model for Korean Hanwoo steer beef. Meat Sci..

[243] Yilmaz M.T., Karakaya M., Aktas N. (2010). Composition and thermal properties of cattle fats. Eur. J. Lipid Sci. Technol..

[244] Knothe G., Dunn R.O. (2009). A comprehensive evaluation of the melting points of fatty acids and esters determined by differential scanning calorimetry. J. Am. Oil Chem. Soc..

[245] Pitchford W.S., Deland M.P.B., Siebert B.D., Malau-Aduli A.E.O., Bottema C.D.K. (2002). Genetic variation in fatness and fatty acid composition of crossbred cattle. J. Anim. Sci..

[246] Woods W.B., Fearon A.M. (2009). Dietary sources of unsaturated fatty acids for animals and their transfer into meat, milk and eggs: A review. Livest. Sci..

[247] Yang A., Larsen T.W., Smith S.B., Tume R.K. (1999). Delta (9) desaturase activity in bovine subcutaneous adipose tissue of different fatty acid composition. Lipids.

